# Enhancing solubility and stability of sorafenib through cyclodextrin-based inclusion complexation: *in silico* and *in vitro* studies†

**DOI:** 10.1039/d3ra03867j

**Published:** 2023-09-11

**Authors:** Aamir Aman, Saba Ali, Panupong Mahalapbutr, Kuakarun Krusong, Peter Wolschann, Thanyada Rungrotmongkol

**Affiliations:** a Program in Bioinformatics and Computational Biology, Graduate School, Chulalongkorn University Bangkok 10330 Thailand t.rungrotmongkol@gmail.com; b Center of Excellence in Structural and Computational Biology, Department of Biochemistry, Faculty of Science, Chulalongkorn University Bangkok 10330 Thailand; c Department of Biochemistry, Center for Translational Medicine, Faculty of Medicine, Khon Kaen University Khon Kaen 40002 Thailand panupma@kku.ac.th; d Institute of Theoretical Chemistry, University of Vienna 1090 Vienna Austria

## Abstract

Sorafenib (SOR) is an oral multikinase inhibitor that effectively hampers the growth and spread of cancer cells by targeting angiogenesis and proliferation. However, SOR tablets (Nexavar) have limited oral bioavailability, ranging from 38% to 49%, due to their low water solubility. To address this issue, cyclodextrins (CDs), widely used to enhance the solubility and stability of lipophilic drugs by encapsulating them within their molecular structure, were considered in this study. We focused on β-cyclodextrin (βCD) and its derivatives, including hydroxypropyl-β-cyclodextrin (HPβCD), dimethyl-β-cyclodextrin (DMβCD), sulfobutylether-β-cyclodextrin (SBEβCD), and compared them with γ-cyclodextrin (γCD) for generating inclusion complexes with SOR. The 200 ns molecular dynamics simulations revealed that SOR could form inclusion complexes with all CDs in two possible orientations: pyridine group insertion (P-form) and chlorobenzotrifluoride group insertion (C-form), primarily driven by van der Waals interactions. Among the four βCD derivatives studied, SOR exhibited the highest number of atom contacts with SBEβCD and demonstrated the lowest solvent accessibility within the hydrophobic cavity of SBEβCD. These findings correlated with the highest binding affinity of SOR/SBEβCD complex determined by SIE, MM/GBSA, and MM/PBSA methods. Experimental results further supported our computational predictions, in which SBEβCD exhibited a stability constant of 940 M^−1^ at 25 °C, surpassing βCD's stability constant of 210 M^−1^. Taken together, our results suggest that the modified CDs, particularly SBEβCD, hold promising potential as an efficient molecular encapsulating agent for SOR, offering improved solubility and stability for this lipophilic drug.

## Abstract 2

Amongst all inclusion complexes of SOR with CDs, SBEβCD exhibit the highest tendency to encapsulate SOR.

## Graphical abstract

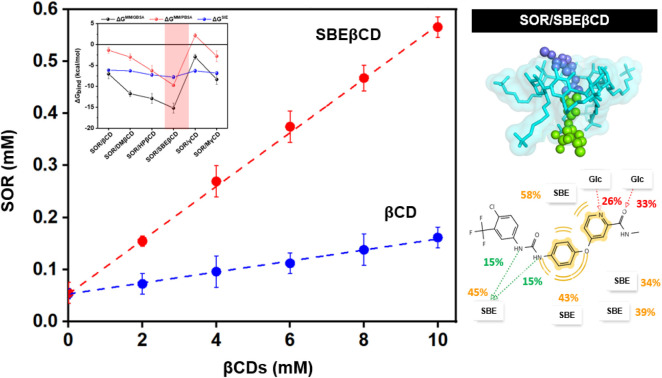

## 1. Introduction

Sorafenib (SOR), also known as 4-(4-(3-(4-chloro-3-(trifluoromethyl)phenyl)ureido)phenoxy)-*N*-methylpicolinamide, is currently the only FDA-approved treatment for hepatocellular carcinoma (HCC). It functions as an oral multikinase inhibitor by targeting several kinases and growth factors, including Raf kinases, vascular endothelial growth factor receptor (VEGFR), Fms-like tyrosine kinase 3 (FLT-3), and c-Kit.^1,2^ Its mechanism of action involves inhibiting angiogenesis, suppressing cell proliferation, and impeding the spread of cancer cells, making it effective against various tumor types.^3,4^ Despite its efficacy, sorafenib has poor water solubility,^5^ which decreases its bioavailability (38–49%) when administered in tablet form, compared to an oral solution.^6^ This limited solubility, combined with its high cytotoxicity to normal tissues, can lead to suboptimal treatment outcomes for many cancer types.^7^ Additionally, the use of sorafenib may give rise to various adverse effects, including dry skin, itching, acne, diarrhoea, hair loss, and vomiting.^8^

Various strategies have been explored to enhance the solubility of poorly water-soluble drugs and substances, including the use of co-solvents, cyclodextrins (CDs) complexation, and nano-engineered delivery systems.^9,10^ Among these approaches, the encapsulation of hydrophobic molecules using CDs has gained significant attention due to its cost-effectiveness, high drug loading capacity, improved bioavailability, commercial availability, favorable pharmacochemical properties, enhanced dissolution rate, and potential to improve the stability of lipophilic compounds, along with excellent biocompatibility.^11,12^ CDs are cyclic oligosaccharides naturally derived from starch through enzymatic breakdown.^13^ They consist of α-d-glucopyranose units connected by α-1,4 glycosidic bonds and possess a cone-shaped structure with a hydrophilic outer surface and a hydrophobic inner cavity. The most commonly encountered natural CDs are αCD, βCD, and γCD, which contain 6, 7, and 8 α-d-glucopyranose units, respectively.^14^ CDs allow guest molecules to enter its nanocavity through van der Waals forces.^15^ βCD derivatives such as sulfobutylether-βCD (SBEβCD) and 2-hydroxypropyl-βCD (HPβCD) offer several advantages over natural βCD. They exhibit higher water solubility, form more substantial complexes with other molecules, and possess lower toxicity, making them more suitable for pharmaceutical applications.^16–19^ Modified forms of βCD, such as methylated βCD (MβCD) and HPβCD, are commonly employed for drug encapsulation due to their ability to form inclusion complexes and their significantly higher water solubility (>500 mg mL^−1^) compared to βCD.^20^ In addition, γCD has been widely utilized in various industries due to its higher water solubility, larger internal cavity, and more bioavailability.^21,22^ Previous studies have demonstrated the significant enhancement of pharmacokinetic, biodistribution, and pharmaceutical properties of regorafenib through the use of mannose-γCD (MγCD).^23^

Despite some existing research on the inclusion complexation of SOR with βCD and γCD,^24^ there is still a lack of knowledge regarding the specific structural details of these complexes. Furthermore, the inclusion complexation of SOR with CD derivatives remains poorly understood. Therefore, the main objective of this study is to enhance our understanding of the structural characteristics of inclusion complexes formed between SOR and six host molecules (βCD, DMβCD, HPβCD, SBEβCD, γCD, and MγCD). All-atom molecular dynamics (MD) simulations and free energy calculations have been employed to investigate the structural dynamics of inclusion complexes in an aqueous solution. Additionally, this work aims to identify the most effective host molecule among the six studied CDs for enhancing the water solubility of SOR. To complement the computational analysis, the solubility and stability of the inclusion complexes were also investigated experimentally.

## 2. Computational details

### 2.1. Preparation of 3D structures of SOR and CDs

The HF/6-31G* level of theory was used to optimize the 3D structure of the SOR using the Gaussian09 (ref. 25) program as per the standard protocol.^26–28^ The protonation state of SOR was characterized using MarvinSketch^29^ at a pH of 7.0. It was discovered that HPβCD with high (7.76) or medium (6.16) degrees of substitution resulted in reduced solubility and increased nephrotoxicity, compared to HPβCD with a low degree of substitution (4.55).^30^ HPβCD with four substitutions on the primary rim (O6) of βCD has been reported to exhibit less probability of cavity self-closure.^31^ Accordingly, this study utilized HPβCD with four HP substitutions on its primary rim. SBEβCD with a degree of substitution of 7 was found to be the most effective host for rasagiline among the different degrees of substitution of SBEβCD evaluated.^32^ The 3D structures of βCD, γCD, and HPβCD were obtained from previous studies.^31,33,34^ The MγCD with a single mannose substitution on the primary rim of γCD was constructed, as reported previously.^23^

### 2.2. Molecular docking study

The CDOCKER module in Accelrys Discovery Studio 2.5 (Accelrys Software Inc., San Diego, CA, USA) was used to generate the inclusion complex between SOR and CDs. The binding affinity of SOR towards CDs was evaluated by conducting a docking process using a 10 Å sphere. From the top 100 hits, their percentage of docked conformations (%DCs) was recorded, and the docked complexes with the lowest binding interaction energy were selected as the starting structures for further MD simulations.

### 2.3. Molecular dynamics simulations

The Glycam-06 (ref. 35) and the general AMBER force fields^36^ were employed to simulate the behavior of CDs and SOR, respectively. Water molecules (TIP3P) were added to the model, with a 15 Å spacing distance, to solvate the SOR/CD complexes. The added water was minimized using a combination of 1000 steps of steepest descent and 3000 steps of conjugated gradient. The entire model underwent an overall minimization using the same methods. The simulations assumed periodic boundary conditions and a 2 fs time step. Initially, the complexes were heated from 10 K to 298 K for 100 ps, followed by three individual all-atom molecular dynamics simulations in the NPT ensemble, with a temperature of 298 K and pressure of 1 atm, utilizing the AMBER20 (ref. 37) software package. The SHAKE algorithm^38^ was applied to constrain hydrogen-involved bonds, and the Particle Mesh Ewald^39^ method was used with a cutoff of 12 Å to calculate charge–charge interactions. Three replicates of all-atom MD simulations (MD#1–3) were conducted for each system, with a duration of 200 ns. To assess the stability of the system, the root-mean-squared displacement (RMSD) was calculated, and the last 100 ns of simulation data was selected for further analyses. The preferred binding orientation of each complex was determined by analyzing the distances between the SOR components and CDs. The most representative structures of the inclusion complex were identified through RMSD clustering and the use of DBSCAN density-based clustering algorithms.^40^ The number of contacts between SOR and CDs was determined using a cutoff value of 3.0 Å. The accessibility of water to SOR was analyzed by calculating the solvent-accessible surface area (SASA) of the complex. The potential energy surface (PES) was calculated to gain insight into the structure of CDs during the simulation. Binding affinity was assessed through solvated interaction energy (SIE),^41^ molecular mechanics/generalized Born surface area (MM/GBSA) and mechanics/Poisson–Boltzmann surface area (MM/PBSA) based binding free energy calculations.^42^

## 3. Experimental studies

### 3.1. Materials

SOR was purchased from Sigma-Aldrich (St. Louis, MO, USA). βCD was obtained from FUJIFILM Wako Pure Chemicals Corporation, Osaka, Japan, while SBE7βCD with a degree of substitution (DS) ranging from 6.0 to 7.1 was acquired from Medchem Express (Monmouth Junction, NJ, USA).

### 3.2. Phase solubility study

The previously reported methods by Higuchi and Connors^43^ were used to conduct a phase solubility study to analyse the host–guest behaviors at 25 °C. An excess amount of SOR prepared in pure water was added to different concentrations of βCD and SBEβCD, ranging from 0 to 10 mM, followed by vortexing and sonication to ensure consistent mixing of components throughout. Note that the samples were unbuffered. It is worth noting that light protection was necessary during the preparation of SBE7βCD and SOR solutions in water. The mixtures were incubated for 72 hours at 25 °C with constant shaking at 250 rpm. Following the incubation, the suspension was subjected to centrifugation at 12 000 rpm for 15 minutes. The resulting saturated supernatants were filtered through 0.45 μm membrane filters to remove any undissolved SOR. The filtrate was then appropriately diluted with a 1 : 1 (v/v) mixture of ethanol and water. The solution's SOR concentration was then measured at 260 nm. The stability constant (*K*_c_) for encapsulation was determined using the formula:
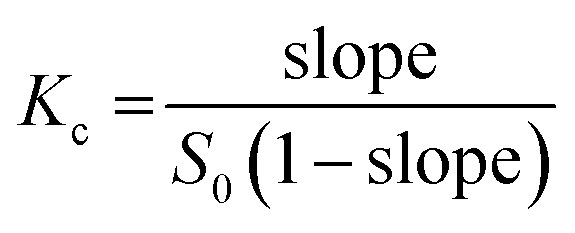
where *S*_0_ is the *y*-intercept indicating how much SOR can dissolve in water without any βCDs.^44^ The experiment was performed three times independently, and the findings were reported as mean ± standard error of the mean (SEM).

## 4. Results and discussion

### 4.1. Binding patterns of SOR inside the cavity of CDs

**Fig. 1:**
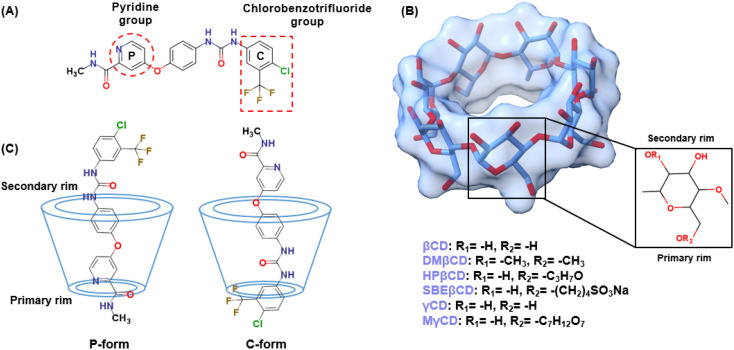
(A) Chemical structure of SOR containing pyridine group and chlorobenzotrifluoride group. (B) 3D structure of all studied CDs. (C) The possible orientations of SOR in complex with CDs: pyridine group insertion (P-form) and chlorobenzotrifluoride group insertion (C-form).

**Fig. 2:**
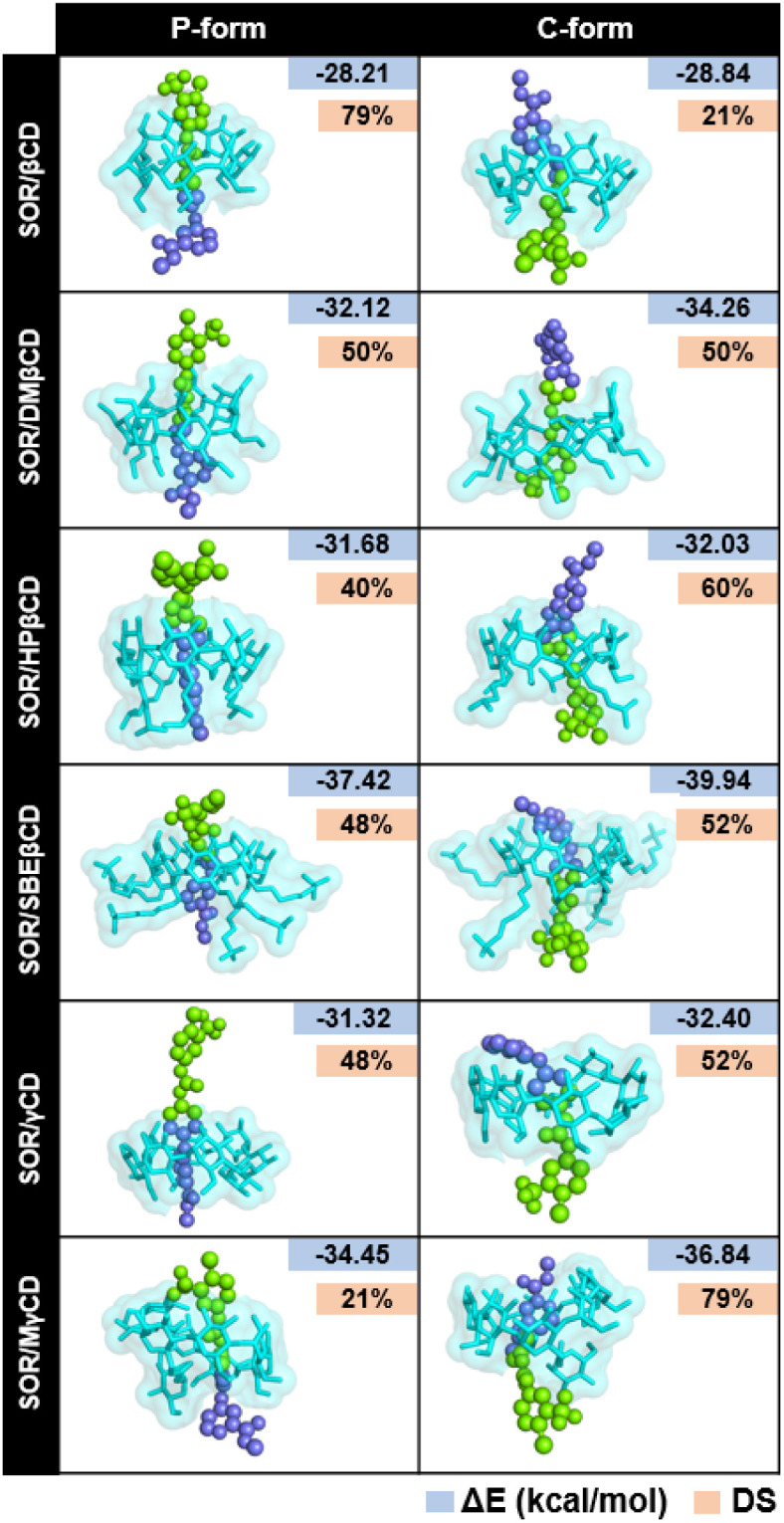
%Docked conformations (%DCs) and CDOCKER interaction energy (Δ*E*) of all docked complexes.

Based on 100 docking runs, it was discovered that SOR had two favorable positions within the hydrophobic interior of CDs. The insertion of the pyridine group of SOR into the CDs' cavity was referred to as the P-form, while chlorobenzotrifluoride group insertion was named as C-form (Fig. 1c). The data in Fig. 2 shows %DCs of P-form and C-form which highlights that the C-form is more prevalent in all complexes compared to the P-form. This finding agrees well with the previously reported 2D NOESY spectra of SOR/βCD and SOR/γCD, where SOR/βCD displayed correlations between the –O2H and H2 protons of βCD and the protons on the ureido group (C-form) of SOR.^24^ In other reported study, in the most stable complex, the fluorine atoms are oriented towards the hydrophobic primary rim of amphiphilic cyclodextrin (aCD) while the oxygen-rich portion of the SOR faces the hydrophilic secondary rim.^45^ C. Phan *et al.* also confirmed the encapsulation of SOR with CDs, where they recorded the ^1^H-NMR spectrum which indicates signals in the region of 9.5–7.0 ppm for the SOR protons and the signals in the range of 5.5–3.0 ppm to the intrinsic proton peaks of SOR/βCD and SOR/γCD.^24^ It is noteworthy, that the signal intensity of the protons in SOR was smaller than that in CDs, which may be due to the modification or shielding in the cavities of CDs after complexation.^24^ The interaction energies of both P-form (ranging from −28 to −37 kcal mol^−1^) and C-form (ranging from −28 to −39 kcal mol^−1^) are similar; thus, both orientations were selected as the starting structures for MD simulations.

### 4.2. System stability

**Fig. 3:**
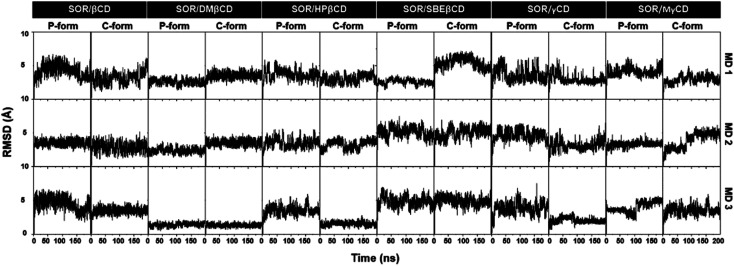
All-atom RMSD profiles of all complexes in both P-form and C-form, plotted over 200 ns in three replicates (MD1-3).

The stability of the inclusion complexes was evaluated by calculating the all-atom RMSD (Fig. 3). Results showed that most complexes remained stable during the simulation, with fluctuations in RMSD ranging from 2–6 Å, while a few systems got stable after 100 ns. Previous study shows that RMSD maps of MD simulation ranging from 1 Å to 4 Å, indicating the majority of the complex formations.^45^ Based on the fact that all inclusion complexes reach equilibrium after 100 ns, further analyses were conducted using the last 100 ns (100–200 ns) of MD simulations.

### 4.3. SOR mobility inside the CD cavity

**Fig. 4:**
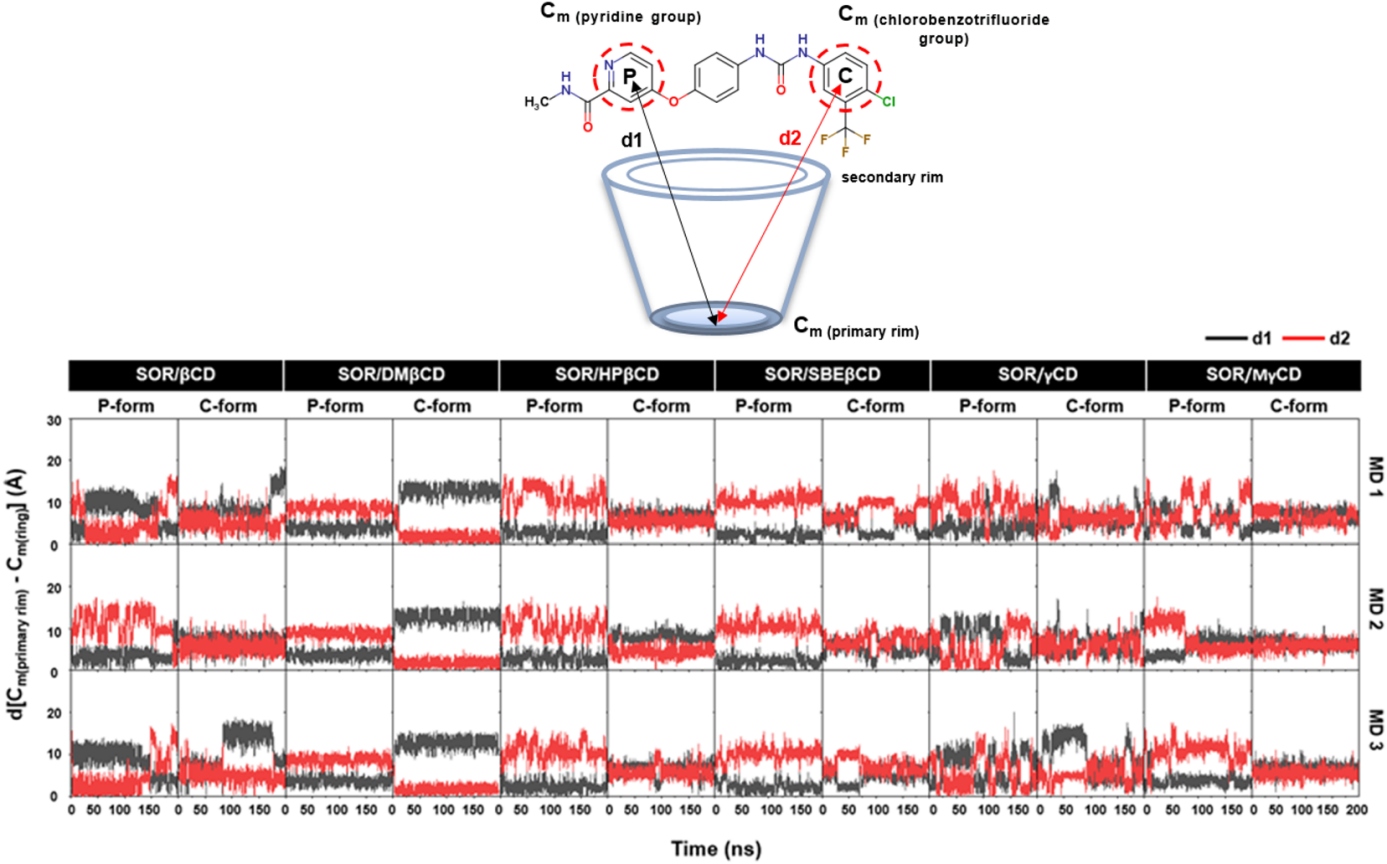
Distance analysis of all inclusion complexes plotted over 200 ns, where *d*[C_m_ (primary rim) − C_m_(P)] (*d*_1_) and *d*[C_m_ (primary rim) − C_m_(C)] (*d*_2_) are represented in black and red, respectively.

The dynamic behavior of the encapsulated SOR inside the hydrophobic interior of CDs was analyzed over time by calculating the distance between the center of mass of pyridine group, ring of chlorobenzotrifluoride group of SOR and the center of mass of the primary rim of CDs, ignoring the functional substituents (Fig. 4). In the case of SOR/βCD, SOR/γCD, and SOR/MγCD in both orientations (P-form and C-form), SOR moves towards the primary rim and secondary rim throughout the simulation because of the flexible pyridine group and the larger size of SOR. Considering SOR/SBEβCD (C-form) inclusion complex, SOR moves towards the narrow rim of SBEβCD and remains inside the side chains of SBEβCD for all three MD simulations. All complexes were further confirmed by taking snapshots of each complex throughout the simulation, as illustrated in Fig. S1–S3 in ESI.†

**Fig. 5:**
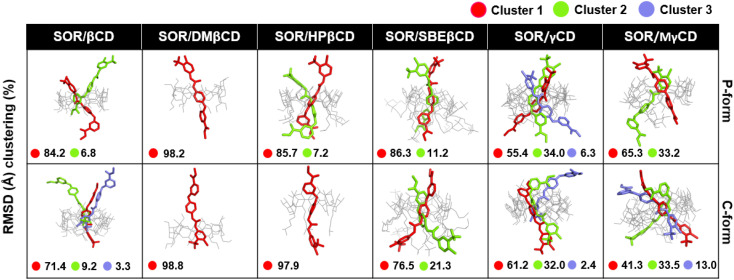
Representative structures of host–guest inclusion of each RMSD cluster for both P-form and C-form of SOR/CDs inclusion complexes. Clusters population was labelled in a percentage.

To verify the results, the RMSD clustering was performed by combining the final 100 ns trajectories of all three MD simulations, based on 10 000 snapshots, depicted in Fig. 5. The results revealed that there were three groups of inclusion complexes, including cluster 1 (red) being the most prevalent for all studied systems, cluster 2 (green) was the second largest population, and cluster 3 (purple) was the lowest population. It was observed that the distribution of populations among the clusters was significantly distinct. In the case of SOR/βCD, SOR/γCD, and SOR/MγCD, all three clusters were found and the remaining complexes only showed two clusters. These outcomes justify the distance analysis results (Fig. 4), specifically in the case of SOR/SBEβCD (C-form) which apparently indicated to be oriented as P-form. Fig. 5 also clarifies that SOR was consistently located within the core of CDs in all complexes, indicating the formation of inclusion complexes.

### 4.4. Atomic contacts and solvent accessibility toward inclusion complex

**Fig. 6:**
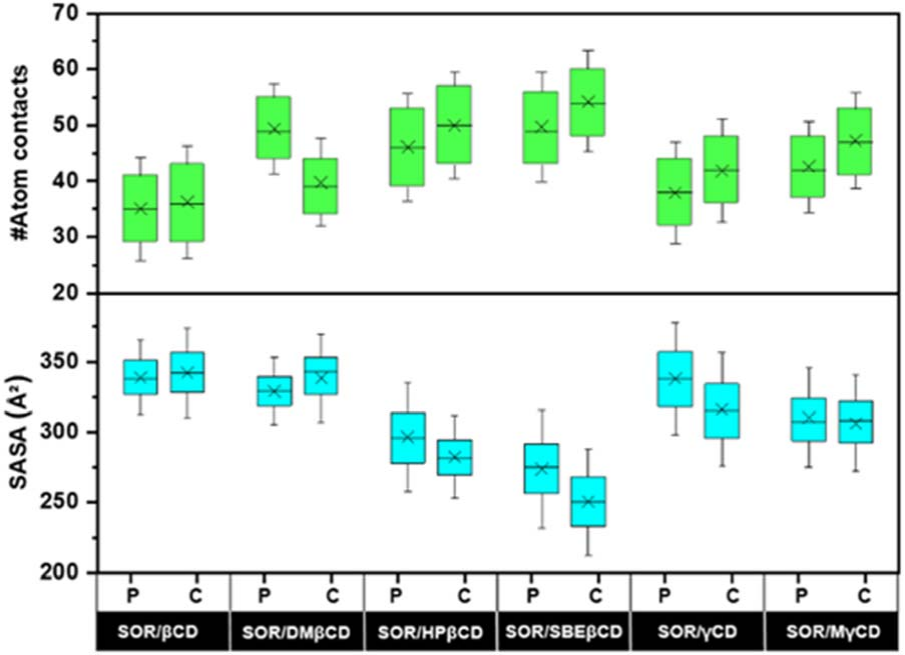
The number of atomic interactions and SASA calculated over last 100 ns for all inclusion complexes. Green and cyan boxes cover the area between the 25th and 75th percentiles. Mean values are represented by a cross, while whiskers determine the standard deviation.

To investigate the encapsulation of SOR by CDs through MD simulations, the number of atomic contacts between SOR and CDs using a cutoff of 3 Å distance was determined (Fig. 6). A high level of molecular contact indicates a favorable and close interaction between the host and guest molecule, while a low level of contact indicates a weaker interaction.^46^Fig. 6 highlights that the average atom contacts of the SOR/SBEβCD inclusion complex (C-form) were the highest (54.2 ± 9.1) among all studied complexes followed by C-form of SOR/HPβCD (50.0 ± 9.6). The second most atom contacts were observed in the P-form of SOR/SBEβCD (49.7 ± 9.8) followed by the P-form of SOR/DMβCD (49.3 ± 8.0). Table S1 in ESI† shows the interactions of SOR with CDs, which is in favor of the statement that SBEβCD could be the best encapsulating agent for SOR.

To validate these results, we further investigate the water accessibility towards SOR (Fig. 6). The idea is that when SOR creates an inclusion complex with CDs, the amount of water molecules surrounding the SOR decreases and the interaction between the host and guest molecule increases. The lowest SASA value of 250.4 ± 25.3 Å^2^ was observed in C-form of SOR/SBEβCD inclusion complex followed by P-form of SOR/SBEβCD (273.7 ± 27.9 Å^2^). The second lowest SASA value was seen in the C-form of SOR/HPβCD (282.2 ± 19.4 Å^2^), followed by the P-form of SOR/HPβCD (296.4 ± 25.6 Å^2^). The highest SASA values were found in the P-form of SOR/βCD (338.9 ± 17.8 Å^2^) and the C-form of SOR/βCD (342.3 ± 21.2 Å^2^), which indicates that SOR/βCD is more likely to interact with water molecules as compared to other complexes. Table S1 in ESI† clearly represent as the SASA values decreases, atom contacts between SOR and CDs increases.^47^ In conclusion, low SASA values support that SBEβCD could be the best host molecule for SOR.

### 4.5. CDs conformations in relation to PES calculations

The evaluation of the structural distortions in CDs upon the binding of SOR was performed through PES analysis. This analysis was based on the measurement of two distances: (1) the distance between adjacent oxygen atoms at the wider rim *d*_*n*_[O3_*n*_ − O2_*n*+1_], and (2) the distance between adjacent glucopyranose units *d*_*n*_[O4_*n*_ − O4_*n*+1_]. These distances were used to determine the strength of the intramolecular hydrogen bonds and the ellipticity of the CDs, respectively. Eqn (1) was applied to calculate probability distributions of these distances and get the value of free energy, *F*(*x*,*y*):

1 *F*(*x*,*y*) = −*K*_B_*T* log[*P*(*x*,*y*)]

where *k*_B_ is the Boltzmann constant, *T* is the absolute temperature (298 K), and *P*(*x*,*y*) is the probability of *x* for *d*_*n*_[O3_*n*_ − O2_*n*+1_] and *y* for *d*_*n*_[O4_*n*_ − O4_*n*+1_] distances.

**Fig. 7:**
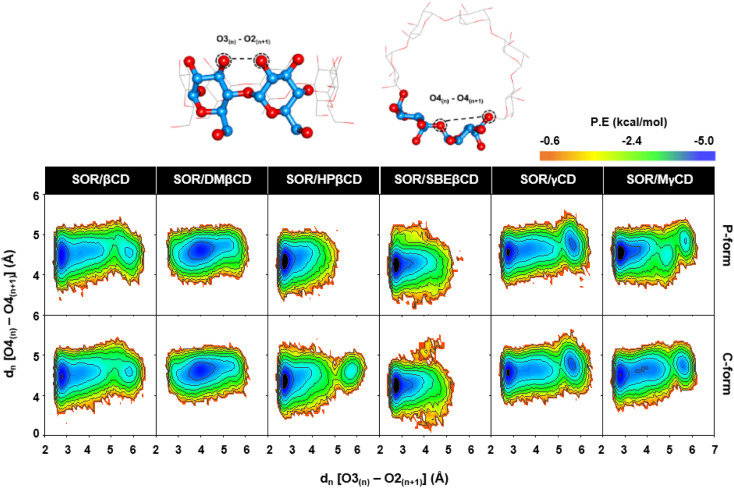
PES plots generated for all studied CDs in complex with SOR, calculated over last 100 ns.

In the PES study of complexes, two local minima (M1 and M2) were observed, as depicted in Fig. 7. The contour graphs display the probability values for distances, colors ranging from red to blue, and darker shades of blue indicating lower free energy level. The encapsulation process resulted in a stable conformation of CDs, which was demonstrated by the presence of a distinct M1 region in all analyzed complexes. This stability was achieved due to the formation of hydrogen bonds between O3_*n*_ and O2_*n*+1_. The M2 region found in SOR/βCD, SOR/γCD, and SOR/MγCD in both P and C-form indicates the distortion of the glucopyranose structures but totally disappeared in the remaining complexes, indicating the most stable complexes. The Intensity of M2 region of SOR/γCD and SOR/MγCD is clearly increased when compared to SOR/βCD, which is also reported previously that the network of hydrogen bonds of O3–O2 in γCD is relatively less than βCD due to the distortion of glucopyranose structures in γCD.^48^ The molecular encapsulation of SOR towards these CDs clearly indicates the formation of hydrogen bonds (higher proportion of O3_(*n*)_ − O2_(*n*+1)_ with a distance of 2–3 Å) on the winder rim, which are in good agreement with the previous studies.^49–51^

### 4.6. System compactness

**Fig. 8:**
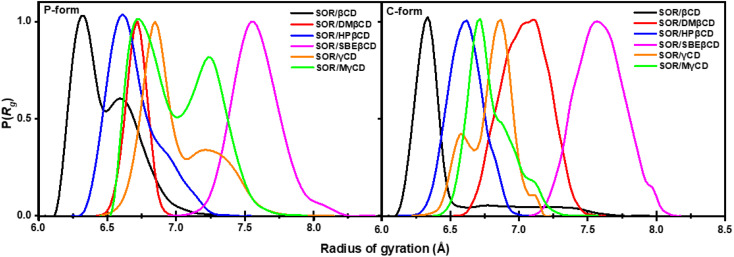
Probability of the radius of gyration of all inclusion complexes both in P-form and C-form.

To gain further insight into the compactness of the system, we calculated radius of gyration (*R*_g_). The collapse in specific structures, such as polymers, proteins, and micelles formation, can be measured by means of *R*_g_.^52^ The probability of the *R*_g_ of all complexes is shown in Fig. 8. In previous study, the average *R*_g_ value of DMβCD, HPβCD, and SBEβCD was 6.60–7.00, 6.85, and 8.03 Å, respectively.^31,50,53^ The average *R*_g_ value of SOR/βCD, SOR/DMβCD, SOR/HPβCD, SOR/SBEβCD, SOR/γCD, and SOR/MγCD was 6.40 ± 0.40, 6.70 ± 0.70, 6.61 ± 0.18, 7.59 ± 0.17, 7.01 ± 0.20, and 7.00 ± 0.30 Å, respectively. The decline in *R*_g_ values observed in the studied complexes are due to the interaction between SOR and CDs, which results in a decreased flexibility of the CDs' conformation upon inclusion complex formation. *R*_g_ values of P-form and C-form were not significantly different from each other. We also found the fluctuation in SOR/βCD (P-form), SOR/γCD, and SOR/MγCD in both P-form and C-form which is due to the distortion of glucopyranose structures of CDs. These findings also support the PES calculation results (Fig. 7). In general, the investigation of molecular compactness provides additional evidence for the formation of an inclusion complex between SOR and CDs.

### 4.7. Binding free energy calculation

To assess the binding strength between host and guest molecules in various inclusion complexes, 10 000 snapshots taken from the last 100 ns MD simulations were utilized for SIE calculations using sietraj,^41^ depicted in Table 1. Compared to other complexes, the value obtained for SOR/SBEβCD (−7.17 to −7.84 kcal mol^−1^) was the lowest. The observed energy value for the SOR/γCD complex (−6.16 to −6.50 kcal mol^−1^) was lower than that of the SOR/βCD complex (−5.80 to −6.24 kcal mol^−1^), which aligns with the previous study where SOR/γCD shows higher stability constant (*K*_C_) value (172.50 M^−1^) than SOR/βCD (68.30 M^−1^).^24^

**Table 1:** Predicted Δ*G*^SIE^ of SOR in complex with all studied CDs, *n* = 3

	P-form			C-form		
	MD1	MD2	MD3	MD1	MD2	MD3
SOR/βCD	−6.14 ± 0.34	−6.24 ± 0.41	−6.11 ± 0.35	−6.19 ± 0.54	−6.13 ± 0.35	−5.80 ± 0.51
SOR/DMβCD	−6.69 ± 0.32	−6.66 ± 0.33	−6.68 ± 0.32	−6.35 ± 0.34	−6.30 ± 0.32	−6.28 ± 0.30
SOR/HPβCD	−6.85 ± 0.43	−6.79 ± 0.41	−6.89 ± 0.43	−7.26 ± 0.41	−7.40 ± 0.41	−7.14 ± 0.41
SOR/SBEβCD	−7.23 ± 0.41	−7.17 ± 0.38	−7.24 ± 0.37	−7.78 ± 0.39	−7.84 ± 0.44	−7.74 ± 0.48
SOR/γCD	−6.16 ± 0.42	−6.30 ± 0.59	−6.25 ± 0.37	−6.50 ± 0.43	−6.25 ± 0.45	−6.29 ± 0.50
SOR/MγCD	−6.58 ± 0.44	−6.97 ± 0.39	−6.60 ± 0.35	−6.92 ± 0.38	−6.84 ± 0.35	−6.65 ± 0.58

**Fig. 9:**
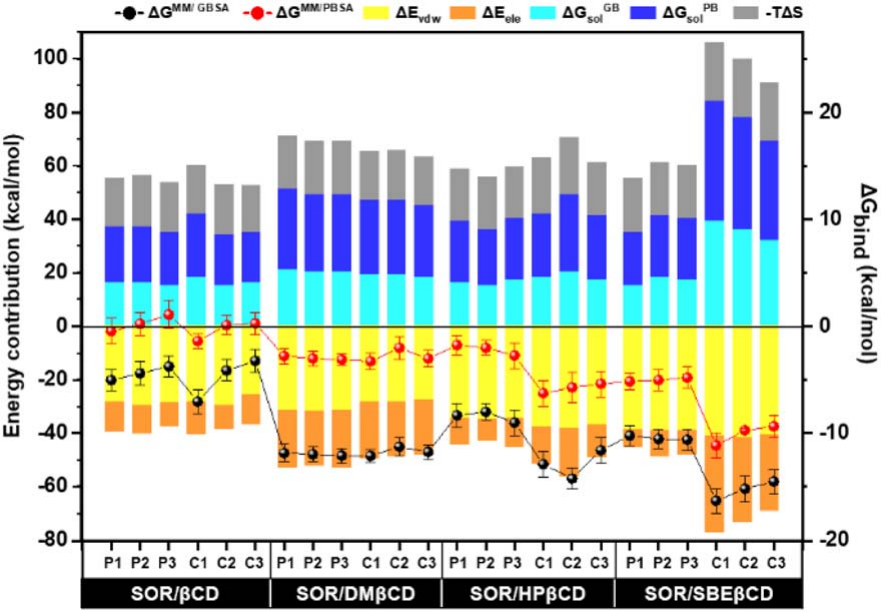
Binding free energy calculation based on the MM/GBSA and MM/PBSA methods and their energy contribution of SOR in complex with CDs, calculated over last 100 ns simulation, *n* = 3.

In order to conduct a more in-depth study of βCD and its derivatives, binding free energy (Δ*G*_bind_) was determined using MM/GBSA and MM/PBSA-based calculations. All energy components are shown in Fig. 9. The formation of inclusion complexes was predominantly influenced by the van der Waals force, which resulted in energy values of approximately −25 to −29 kcal mol^−1^ for SOR/βCD, −27 to −31 kcal mol^−1^ for SOR/DMβCD, −34 to −38 kcal mol^−1^ for SOR/HPβCD and −38 to −41 kcal mol^−1^ for SOR/SBEβCD respectively. These findings align with previous studies that have highlighted the critical role of the van der Waals force in driving the formation of inclusion complexes.^54,55^ The calculated Δ*G*^MM/GBSA^ for all complexes can be ranked as SOR/SBEβCD > SOR/HPβCD > SOR/DMβCD > SOR/βCD, with the value of −10.22 ± 0.97 to −16.32 ± 1.10 kcal mol^−1^, −7.99 ± 0.80 to −14.21 ± 1.10 kcal mol^−1^, −11.26 ± 0.90 to −12.12 ± 0.59 kcal mol^−1^, and −3.22 ± 1.10 to −7.02 ± 1.10 kcal mol^−1^ respectively. The Δ*G*^MM/PBSA^ exhibited the same trend as Δ*G*^MM/GBSA^ with the value of −4.79 ± 1.00 to −11.14 ± 1.10 kcal mol^−1^, −1.75 ± 0.90 to −6.25 ± 1.20 kcal mol^−1^, −2.00 ± 1.10 to −3.00 ± 0.70 kcal mol^−1^ and 1.00 ± 1.10 to −1.00 ± 0.70 kcal mol^−1^ respectively. Studies have provided evidence that SBEβCD exhibits greater inclusion capacity than parent βCD because of the long hydrocarbon chain and hydrophobic butyl moiety present in its structure, which increases its hydrophobicity.^56,57^

**Fig. 10:**
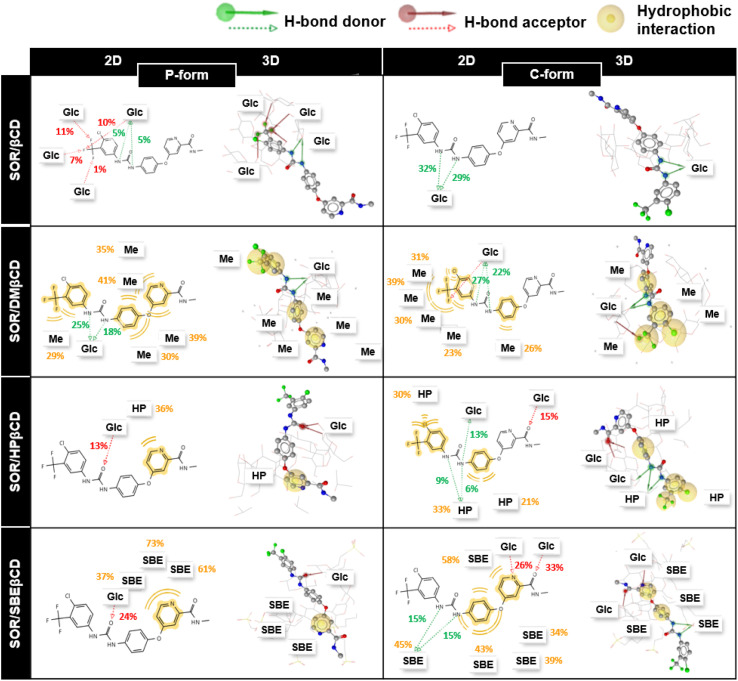
The 2D and 3D guest–host interactions of βCDs in both orientations (P-form and C-form). Glc, Me, HP and SBE represent glucopyranose unit, methyl, hydroxypropyl, and sulfobutylether substitutions, respectively.

In addition, to understand the interactions of SOR with βCD and its derivatives, their percentage of interactions was calculated for the last 100 ns using LigandScout.^58^Fig. 10 highlighted the contribution of individual glucopyranose units or substituted groups toward SOR. It was found that the interactions of SOR/SBEβCD complex in both P-form and C-form were higher (up to 73% in the case of hydrophobic interactions and up to 33% in the case of hydrogen bonding) than SOR/HPβCD in both P-form and C-form (hydrophobic interaction; 36% and hydrogen bonding; 15%), SOR/DMβCD in both P-form and C-form (hydrophobic interaction; 41% and hydrogen bonding; 27%) and SOR/βCD in both P-form and C-form (hydrogen bonding; 32%), which is consistent with Δ*G*_bind_ calculations (Fig. 9). When interacting with substituted groups on βCD, SOR exhibits hydrophobic interactions, while with the glucopyranose units of βCD, it forms hydrogen bonds.

### 4.8. Phase solubility study

**Fig. 11:**
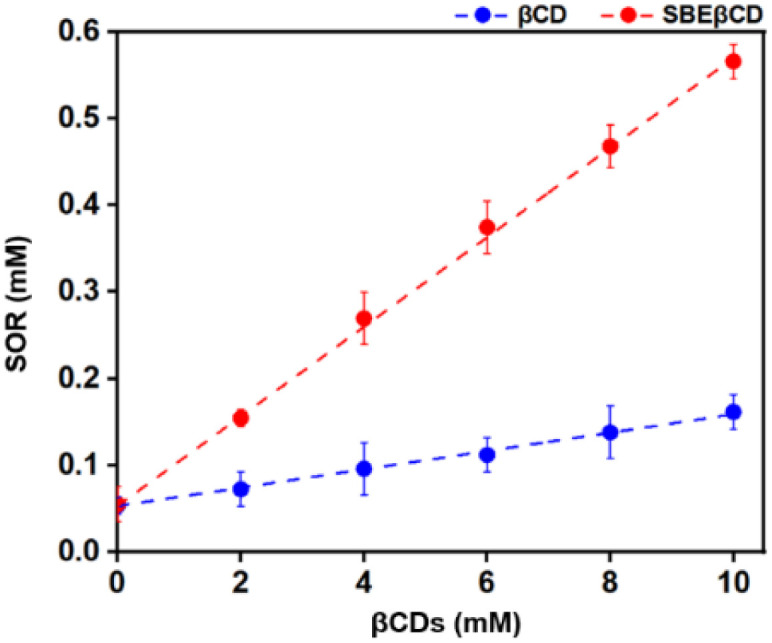
The solubility behavior of the complex formed between SOR and βCDs at 25 °C as shown in the phase solubility plot.

The interaction between SOR and βCDs was analyzed experimentally using a phase solubility diagram where the concentration of βCDs was plotted along the *x*-axis and SOR concentration on *y*-axis. The results (Fig. 11) revealed that the concentration of SOR increased linearly with the increasing concentration of CDs. This finding suggests that SOR and the two CDs, βCD and SBEβCD can form inclusion complexes in a 1 : 1 stoichiometry ratio between the host and guest acquiring A_L_ type plot.^24^ Previous studies have demonstrated that CDs can increase the stability of several drugs/compounds such as amoxicillin,^59^ baicalein,^60^ dicloxacillin,^61^ and eugenol.^62^ By formation of inclusion complexes, CDs provide a protective shield around drug molecules, shielding them from environmental factors that could lead to degradation or loss of efficacy.^63^ The effect of complexation on the chemical stability of drugs in terms of stability constant has been reported in previous studies for amoxicillin,^59^ cephalotin,^64^ and doxorubicin.^65^ As shown in Table 2, the stability constant (*K*_c_) of SOR/SBEβCD displays much better than SOR/βCD with the values of 940 and 207 M^−1^, indicating that SBEβCD is a better host than βCD for inclusion complexation with SOR.

**Table 2:** The stability constants (*K*_c_) of SOR/βCD and SOR/SBEβCD determined at 25 °C

	Type	Slope	*R* ^2^	Intercept	*K*_c_ (M^−1^)	Reported *K*_c_ (M^−1^)
SOR/βCD	A_L_	0.011	0.996	0.052	210	68,^24^ 735 (ref. 66)
SOR/SBEβCD	A_L_	0.051	0.999	0.058	940	—
SOR/γCD	B_S_	—	—	—	—	172 (ref. 24)

Similarly, the previous studies reported that the phase solubility curve for SOR and βCD was an A_L_-type, with the *K*_c_ value of 68.3 M^−1^ and 735.8 M^−1^.^24,66^ However, the phase solubility curve for SOR and γCD was reported to be a B_S_-type, with a *K*_c_ value of 172.5 M^−1^.^24^

## 5. Conclusion

In this study, we conducted 200 ns MD simulations to observe the behavior of SOR when bound to CDs. Our findings suggest that there are two potential binding modes (referred to as P-form and C-form) for the SOR-CD complex, as indicated by molecular docking. According to the analysis of water accessibility and atomic contacts, the SBEβCD has higher encapsulation efficiency with SOR than other CDs. PES calculation indicated that all systems have a significant population of stable M1 region, but a distortion of glucose units (M2) was only observed in SOR/βCD, SOR/γCD, and SOR/MγCD systems. In the MM/GBSA and MM/PBSA calculations, SOR/SBEβCD exhibited the lowest Δ*G*_bind_ values, followed by SOR/HPβCD, SOR/DMBCD, and SOR/βCD, respectively. In the analysis of MM energy components, the inclusion complex is primarily formed by the van der Waals interaction. Phase solubility studies indicated that βCD and SBEβCD were able to enhance the solubility of SOR. Moreover, the observed A_L_-type profile implied a 1 : 1 interaction between the host and guest molecules, and a greater *K*_c_ was observed in SOR/SBEβCD. Based on the structural evidence and experimental results, it can be concluded that CDs, particularly SBEβCD, have the potential as effective drug carrier for SOR to enhance its water solubility and stability.

## Conflicts of interest

The authors report no conflict of interest, financial or otherwise.

## Supplementary Material

RA-013-D3RA03867J-s001
