# Supplementary material for: Enhancing solubility and stability of sorafenib through cyclodextrin-based inclusion complexation: in silico and in vitro studies
Source: RSC Adv. 2023 Sep 11;13(39):27244–54. doi: 10.1039/d3ra03867j (PMC10494890; doi:10.1039/d3ra03867j)
Supplement: RA-013-D3RA03867J-s001 [file RA-013-D3RA03867J-s001.pdf]

Electronic Supplementary Material (ESI) for RSC Advances.
This journal is © The Royal Society of Chemistry 2023

Supplementary Data

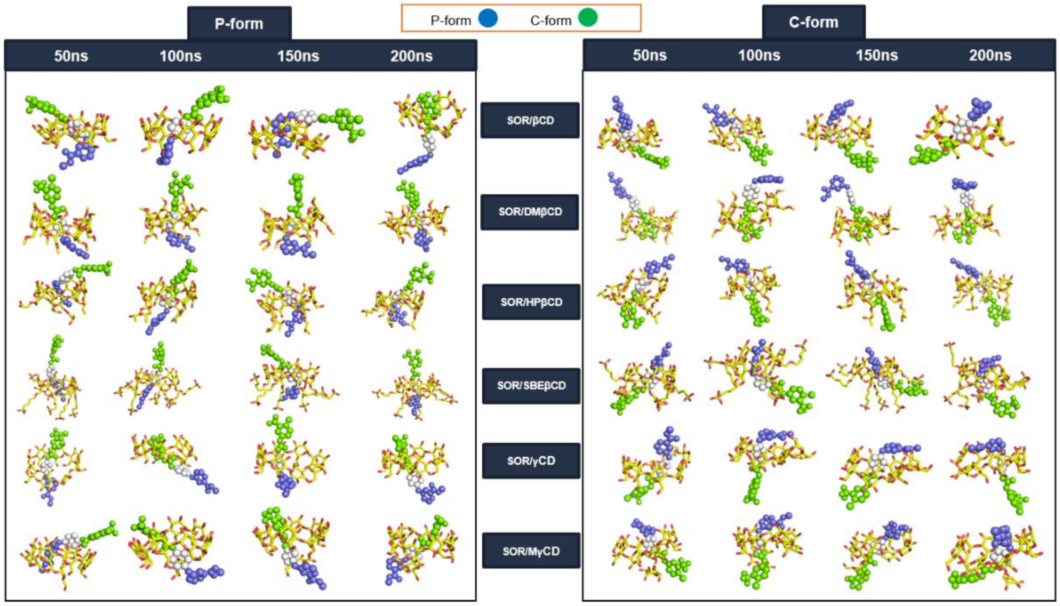

Figure S1. MD1 snapshots of all simulated complexes, taken at 50ns, 100ns, 150ns, and 200ns.

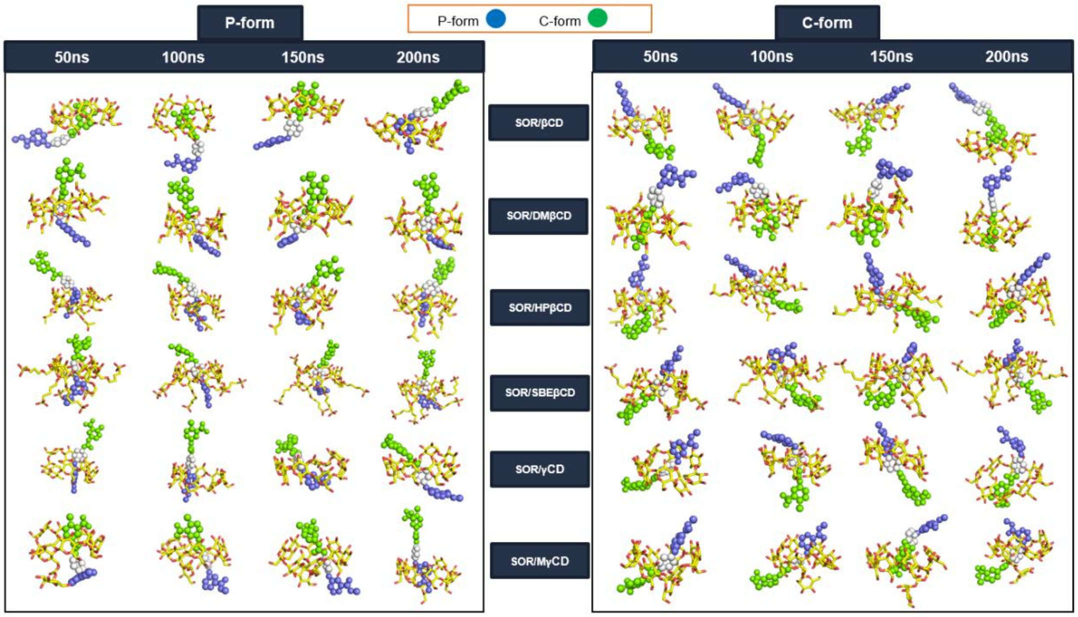

Figure S2. MD2 snapshots of all simulated complexes, taken at 50ns, 100ns, 150ns, and 200ns.

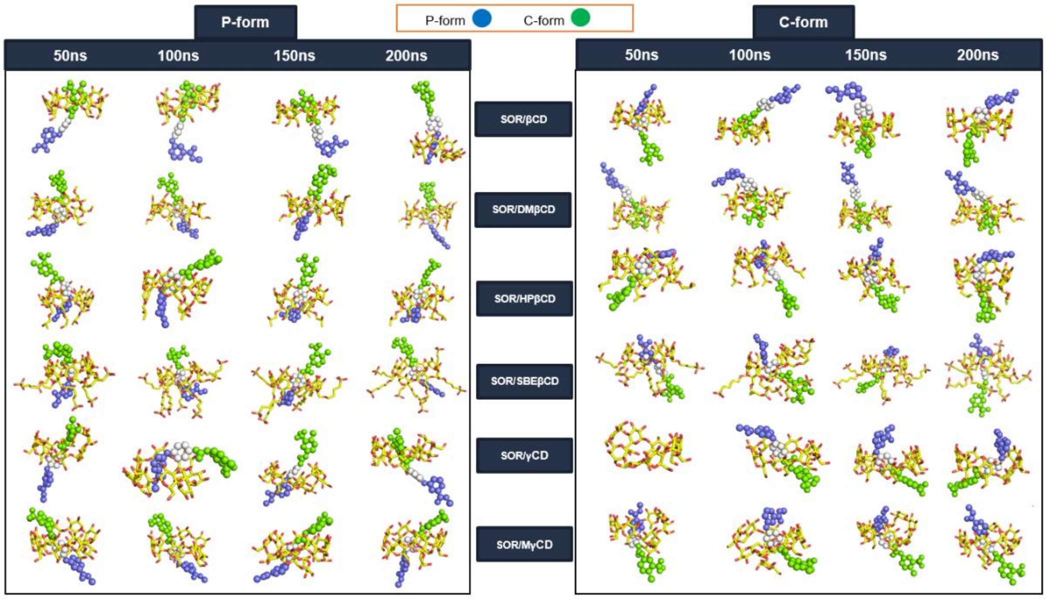

Figure S3. MD2 snapshots of all simulated complexes, taken at 50ns, 100ns, 150ns, and 200ns.

Table S1. Number of contacts and SASA values of all complexes. The data are expressed as mean ± standard deviation, n = 3.

Complexes	Number of contacts		SASA (Å^2)	
	**P-form**	**C-form**	**P-form**	**C-form**
**SOR/βCD**	35.06 ± 9.2	36.29 ± 9.3	338.92 ± 17.8	342.33 ± 21.2
**SOR/DMβCD**	49.33 ± 8.0	39.76 ± 7.8	329.15 ± 15.9	338.51 ± 20.9
**SOR/HPβCD**	46.12 ± 9.6	50 ± 9.6	296.47 ± 25.6	282.23 ± 19.4
**SOR/SBEβCD**	49.73 ± 9.8	54.29 ± 9.1	273.77 ± 27.9	250.44 ± 25.3
**SOR/γCD**	37.89 ± 9.1	41.82 ± 9.3	337.96 ± 26.6	316.5 ± 26.9
**SOR/MγCD**	42.59 ± 8.1	47.28 ± 8.6	310.35 ± 23.4	306.4 ± 22.7
